# miRNA-Mediated Control of B Cell Responses in Immunity and SLE

**DOI:** 10.3389/fimmu.2021.683710

**Published:** 2021-05-17

**Authors:** Stephanie L. Schell, Ziaur S. M. Rahman

**Affiliations:** Department of Microbiology and Immunology, Pennsylvania State University College of Medicine, Hershey, PA, United States

**Keywords:** miRNA, autoimmunity, B cells, germinal center, systemic lupus erythematosus

## Abstract

Loss of B cell tolerance is central to autoimmune diseases such as systemic lupus erythematosus (SLE). As such, the mechanisms involved in B cell development, maturation, activation, and function that are aberrantly regulated in SLE are of interest in the design of targeted therapeutics. While many factors are involved in the generation and regulation of B cell responses, miRNAs have emerged as critical regulators of these responses within the last decade. To date, miRNA involvement in B cell responses has largely been studied in non-autoimmune, immunization-based systems. However, miRNA profiles have also been strongly associated with SLE in human patients and these molecules have proven critical in both the promotion and regulation of disease in mouse models and in the formation of autoreactive B cell responses. Functionally, miRNAs are small non-coding RNAs that bind to complementary sequences located in target mRNA transcripts to mediate transcript degradation or translational repression, invoking a post-transcriptional level of genetic regulation. Due to their capacity to target a diverse range of transcripts and pathways in different immune cell types and throughout the various stages of development and response, targeting miRNAs is an interesting potential therapeutic avenue. Herein, we focus on what is currently known about miRNA function in both normal and SLE B cell responses, primarily highlighting miRNAs with confirmed functions in mouse models. We also discuss areas that should be addressed in future studies and whether the development of miRNA-centric therapeutics may be a viable alternative for the treatment of SLE.

## Introduction

B cell development and function is critical for the establishment of a B cell repertoire that can respond to a diversity of foreign antigens (1). Antigenic exposure initiates B cell responses that target invading pathogens and leads to the formation of long-lived plasma cell and memory B cell responses that protect the host against future reinfection (2). While B cells are critical for the establishment of normal immune responses against pathogens, they can become dysregulated under certain circumstances, leading to the development of autoimmunity (3). Systemic lupus erythematosus (SLE) is a complex autoimmune disease that causes multi-organ dysfunction. The onset of SLE is dependent on both the possession of susceptibility genes and the environmental triggers (e.g. infection, chemicals, retroviral elements) (4, 5). Genetic and environmental factors synergize to cause aberrantly regulated immune activation which leads to the loss of B cell tolerance to self-antigens and high-affinity anti-nuclear antibody (ANA) production (6, 7). ANAs generated by B cells form immune complexes that enter the circulation and deposit in peripheral tissues, leading to the recruitment of myeloid cells, which promote local inflammation (8–10). Inflammation in the kidneys, termed lupus nephritis (10), and various cardiovascular disease manifestations (11) are common causes of morbidity and mortality in individuals living with SLE.

Due to the fact that much still remains unclear about the development of lupus, only one FDA approved therapy specifically developed and approved for SLE, belimumab, has emerged (12). Belimumab is a monoclonal antibody that targets B cell survival by binding to and sequestering Blys, an essential B cell survival factor. Belimumab has an encouraging efficacy in dampening disease manifestations, however it also leaves patients susceptible to infection, as it non-specifically suppresses the immune system (13, 14). A better understanding of the mechanisms involved in SLE development is required to develop novel therapeutics for SLE that may avoid some of the negative immunosuppressive effects of current therapies. The development of microRNA (miRNA) therapeutics has started to gain traction for the treatment of other diseases (15), but their implementation in autoimmunity is still lacking as more studies are required to fully elucidate the contribution of these factors to disease development and progression.

As such, efforts to understand the role of miRNA function in the development of normal B cell responses and dysregulation of these miRNAs in SLE represents a growing field. In general, miRNAs have been implicated as causative agents and biomarkers in a number of diseases (16, 17). In regard to SLE, miRNA centric studies have focused on the differences in miRNA expression between the healthy and diseased states, what cell types are altered by aberrant miRNA expression, and what genes and processes these miRNAs target to either promote or prevent autoimmunity. Many studies have focused on profiling the miRNAs that are expressed in healthy individuals versus those with SLE, with some of these studies determining the miRNAs expressed during the active versus inactive stages of disease (18–23). Cells and tissues used for miRNA profiling in SLE vary, but most studies have profiled the expression of miRNAs in peripheral blood mononuclear cells (PBMCs), B cells, T cells, and blood. Additionally, many studies have assessed miRNAs associated with lupus nephritis through the analysis of urine (24, 25). In addition to profiling miRNAs in human patients, miRNA profiling has been performed in animal models of SLE, demonstrating that there is a conserved profile between several different lupus mouse models and human patients (19, 26, 27).

Broad miRNA profiling has divulged a large amount of information about miRNA expression patterns in normal and SLE B cell responses and has opened the door for mechanistic studies. These mechanistic studies are required to determine how individual or combinations of miRNAs are specifically involved in the loss of B cell tolerance. Additionally, understanding if similar mechanisms are involved in normal protective B cell responses is important for shaping any future therapeutic pursuits. First, we will briefly outline how miRNAs function and the different stages of B cell development and response to antigen. We will then discuss key studies in non-autoimmune and autoimmune systems that frame our understanding of miRNAs in these responses and the implications for therapeutic targeting in the future.

## miRNA Processing and Targeting Mechanisms

miRNAs are small non-coding RNAs, approximately 22 nucleotides in length, that mediate post-transcriptional gene regulation. miRNAs are transcribed from the genome by RNA Polymerase II *via* dedicated promoters or are processed from intronic or exonic sequences located in other transcription units (28–32). This generates a primary miRNA transcript (pri-miRNA). While still in the nucleus, the pri-miRNA is cleaved into the hairpin shaped pre-miRNA by the Microprocessor complex, which contains the RNAse III enzyme Drosha and RNA binding protein DGCR8 (33–36). The pre-miRNA, which is approximately 60-70 nucleotides, is exported from the nucleus into the cytosol *via* the activity of exportin-5 and ran-GTP (37). Once in the cytosol, Dicer cleaves the pre-miRNA into a duplex structure (38, 39). The mature miRNA duplex associates with Argonaute and is dissociated into the 5’ guide strand, which is preferentially retained by Argonaute, and the 3’ passenger (or star) strand, which is preferentially degraded (40). Binding to Argonaute and association with additional proteins that comprise the RNA Induced Silencing Complex (RISC) stabilizes the miRNA from degradation (40–42).

**Figure 1:**
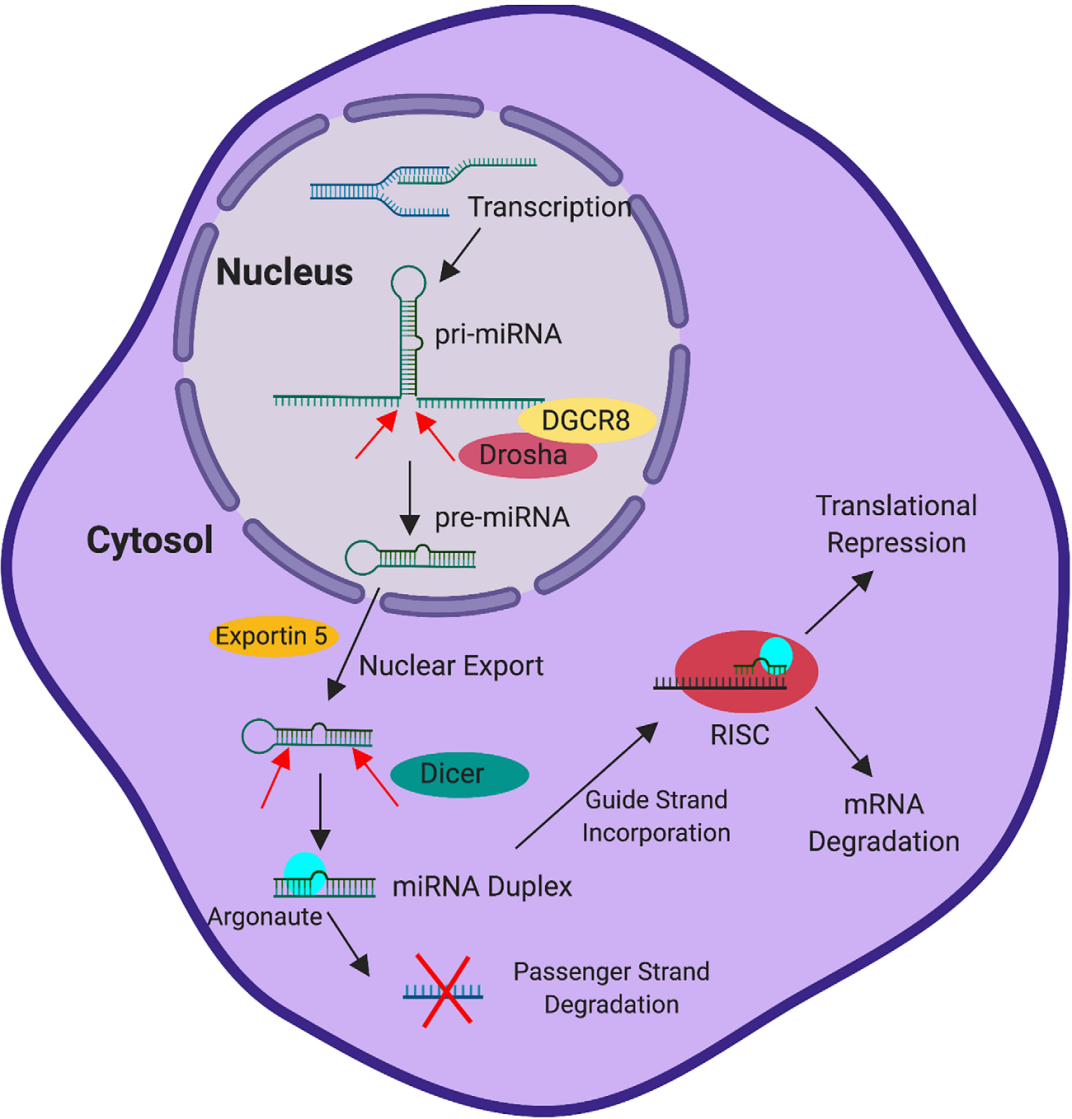
miRNA Processing and Activity. Transcription induced in the nucleus generates a pri-miRNA transcript. The pri-miRNA is cleaved by Drosha, with the aid of co-factor DGCR8, into the pre-miRNA while still in the nucleus. Subsequently, exportin 5 exports the pre-miRNA into the cytoplasm. Following delivery into the cytoplasm, Dicer cleaves the pre-miRNA into the mature miRNA duplex. The mature miRNA duplex (associated with Argonaute) is then dissociated into two strands, the guide strand and the passenger strand. The guide strand preferentially associates with Argonaute in the RNA-induced silencing complex (RISC) and the passenger strand is preferentially degraded. Following association of the miRNA and target transcript, the RISC drives the degradation of the mRNA or mediates translational repression to control gene expression.

Once incorporated into the RISC, the miRNA has been traditionally thought to exert genetic control by base pairing with complementary sequences found in the 3’ UTR of gene transcripts (43–45). However, more recently binding has also been observed within coding regions and 5’ UTRs (44–46). While the 5’ guide strand is typically incorporated into the RISC, the passenger strand can also be incorporated to target its own set of genes, though usually at a reduced level compared to the guide strand (47, 48). The targeting efficiency achieved by the miRNA can depend on the binding strength of the interaction, as miRNA-transcript interaction can occur through perfect or slightly imperfect complementarity with 6-8 base pair motifs located in the target transcript (49). Once the miRNA interacts with its target, negative regulation of gene expression can occur through both degradation of the transcript and translational repression (50–54). Regardless of the regulatory mechanism employed, ultimately the effect is the impediment of protein being translated from target transcripts. This process is summarized in **Figure 1**.

To date, there have been over 1000 miRNAs discovered, with each miRNA capable of targeting hundreds of genes. A significant portion of miRNAs are found in clusters in the genome, further adding to the sophistication with which they can impart genetic control (55). Importantly, miRNAs are highly conserved among species, making their functional study in mouse models relevant to developing an understanding of their function in human (56, 57). In addition to binding sequence and strength of interaction, the ability of a miRNA to target complementary transcripts relies on the level of miRNA expression in the cell type of interest as well as the number and expression level of target genes in the same cell (58). Accordingly, the expression of the miRNAs and target genes vary in different tissues and cell types, and at different stages of development, making miRNA-mediated regulation a fluid process that is extremely specific to conditions and outside stimuli (59). This applies to the immune response where miRNA function is critically important at various stages (60). The profile of miRNAs among immune cell subsets and their functions in these cells confers the ability to specifically fine-tune the activity of many diverse signaling pathways associated with the activation and regulation of immune cell functions. As such, one miRNA can have vastly different gene targets and effects among different immune cell subsets (61). In this review, we focus on how this concept can be applied to miRNA function in normal B cell responses and B cell tolerance in the context of SLE.

## Key Stages of the B Cell Life Cycle in Development and Tolerance

**Figure 2:**
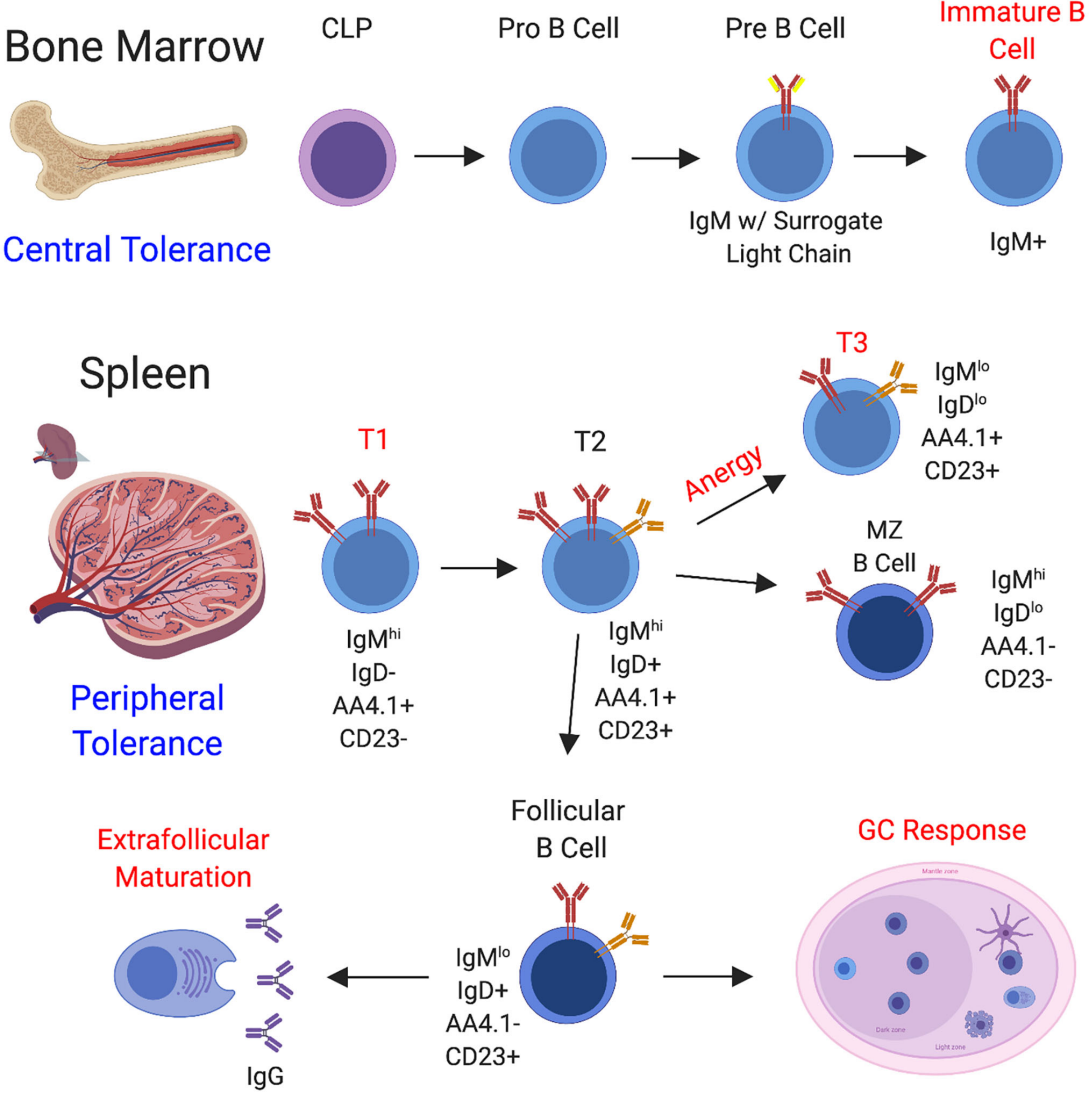
B Cell Development and Sites of B Cell Tolerance. B cell development begins in the bone marrow through a series of steps. Hematopoietic progenitor stem cells (HPSCs) undergo a series of differentiation steps leading to the generation of the common lymphocyte progenitor (CLP), from which B cell differentiation can proceed. Throughout the stages of B cell development in the bone marrow, a functional BCR is assembled and central tolerance is employed, whereby self-reactive B cells are directed to undergo receptor editing or cell death. B cells that pass this checkpoint migrate to the secondary lymphoid organs, including the spleen (depicted here) and lymph nodes. In the spleen, B cells continue to mature and peripheral B cell tolerance is enacted at several different stages of development. B cells enter the spleen at the T1 stage and strong BCR engagement can drive apoptosis at this stage. T1 B cells can progress on to the T2 stage, and from there can be induced to seed the marginal zone and follicle. In addition to regulation at the T1 stage, transitional B cells can be directed to undergo anergy and assume the T3 phenotype, whereby the BCR is downregulated to promote hyporesponsiveness. Marginal zone B cells generally become activated to provide a source of IgM. Alternatively, the follicular B cell subset directs the generation of IgG-secreting plasmablasts through both the follicular germinal center pathway and the extrafollicular pathway. Regulation is employed through both of these pathways. To summarize, well-documented regulatory stages are highlighted in red.

B cell development begins in the bone marrow with commitment of the common lymphocyte progenitor (CLP) to the B cell lineage (62), followed by further differentiation through the stages of pro B cell, pre B cell, and immature B cell (1). In the bone marrow, B cells undergo VDJ recombination to produce a diverse array of BCR specificities and processes exist to negatively select autoreactive B cells that form during this process (central tolerance) (63, 64). Functional, non-autoreactive B cells then egress to the secondary lymphoid organs where they acquire a transitional phenotype. Transitional B cells consist of three independent fractions, the T1 fraction, the T2 fraction, and the T3 fraction (65). Transitional B cells receiving the appropriate levels of stimulation and survival signals eventually differentiate into marginal zone or follicular B cells, whereas autoreactive B cells can be regulated at the T1 and T3 stages through apoptosis or anergy (66, 67) (**Figure 2**). In SLE, defects have been observed in early stages of B cell tolerance and loss of tolerance at this stage is usually linked to the possession of certain genetic susceptibility loci (68–72).

Mature B cells enter two major pathways following antigenic challenge to generate antibody responses, the germinal center (GC) and the extrafollicular pathway. During pathogen-driven immune responses, B cell development through the GC is critical for the generation of plasma cells that secrete high-affinity, class-switched antibodies and the differentiation of memory B cells (73, 74). However, GCs can become enlarged and dysregulated in SLE, leading to the production of high-affinity, class-switched autoantibodies that cause downstream pathology (74, 75). Many reviews have extensively detailed the mechanisms involved in the initiation and maintenance of GC responses driven by foreign antigen and in autoimmunity (73, 74, 76).

Alternatively, extrafollicular foci form in the red pulp and can occur rapidly in response to T-independent and T-dependent antigens (77). Activation of B cells through the extrafollicular pathway leads to rapid plasmablast formation, from which a select number of plasma cells will develop. Responses generated through the extrafollicular pathway can also undergo class-switching and somatic hypermutation independent of the GC, although at a lower frequency (77). In SLE, significant maturation of autoreactive B cells can occur outside of the GC (78–82). Ultimately, dysregulation of B cell responses at any stage of development and response to antigen can lead to autoimmunity.

## miRNA Function in Protective B Cell Responses

While the goal for therapeutic development is to ultimately understand how miRNA expression and function is dysregulated in SLE, in order to achieve this, we must also understand how miRNAs function during normal B cell responses. This is important because miRNA expression level heavily impacts its function. miRNAs may drive aberrant B cell regulation due to overexpression, underexpression, or novel expression in B cells or other cell types that affect B cell responses. Additionally, any potential therapeutic design will ideally leave miRNA function involved in normal B cell responses intact to prevent host susceptibility to infection. The studies that have shaped our current understanding of miRNA function in B cell responses are discussed below. While the focus is predominantly on the B cell and T cell intrinsic expression of these molecules, it is important to note that their expression in innate immune cell types can also shape B cell responses through the regulation of cytokines and other factors.

Technically, studies of miRNA contribution to B cell responses are comprised of multiple approaches which collectively help build a full picture of miRNA involvement. miRNA expression profiling studies establish a starting point by identifying specific miRNAs for further functional analysis. The following phenotypic studies that narrow down on individual miRNAs have implemented a combination of *in vitro* and *in vivo* approaches. While *in vitro* approaches cannot determine the absolute requirement for specific miRNAs in the generation of B cell responses that require specific signals and interactions *in vivo*, such as GC responses, they can identify and confirm mRNA targets in some cell types of interest. On the other hand, mouse models that implement overexpression (lentiviral or genetic), miRNA antagomir administration, or knockouts of individual miRNAs are valuable tools for determining the absolute and non-redundant requirements for these factors in generating specific B cell responses and highlight the function of miRNAs in the presence of stimuli that are specific to *in vivo* conditions (**Tables 1**, **2**).

**Table 1:** Methods to Study or Therapeutically Modulate miRNAs in Mouse Models.

Objective	Methods	Purpose
Global Loss of Function	Knockout mouse models	Absolute, non-redundant function
		Complete loss of expression
Cell Type Specific Loss	Cre-lox systems	miRNA function in a specific cell type
of Function	Bone marrow reconstitution	
Overexpression	miRNA mimics/agomirs (delivery by VLPs, nanoparticles, etc.)	Generate autoimmunity in non-autoimmune backgrounds
	Viral vector expression	Prevent autoimmunity if miRNA is regulatory
Dampened Expression	Antagomirs/LNA anti-miRs	Study effects dependent on expression level
	Viral vector expression of sponge	More therapeutically relevant
	Heterozygous mice (if expression is reduced)	
Temporal Effects	Inducible system (e.g. Dox)	Differentiate prophylactic versus therapeutic effects
	Timed administration of antagomirs/viral vectors	

VLP, virus like particle; LNA, locked nucleic acid; Dox, doxycycline.

**Table 2:** miRNAs and Direct Target Genes in Protective B Cell Responses.

miRNA	Site	Confirmed or Predicted Target Gene(s)	Key Assays Performed	Ref #
miR-15	Bone Marrow	*ccne1* (cyclin E1)	LUC, GEN MOD	(83)
miR-17~92	Bone Marrow	*pten*	LUC, GEN MOD	(84)
miR-23a	Bone Marrow	*bach1*	LUC, GE	(85)
		*runx1*	GE	(85)
		*satb1*	GE	(85)
		*ikzf1* (Ikaros)	GE	(85)
miR-34a	Bone Marrow	*foxp1*	LUC, GEN MOD	(86)
miR-125b	Bone Marrow	*s1pr1*	LUC, 3UTR	(87)
miR-126	Bone Marrow	*irs1*	BP, GE	(88)
miR-128-2	Bone Marrow	*adora2b* (A2B)	LUC, GE	(89)
		*malt1*	LUC, GE	(89)
miR-132	Bone Marrow	*sox4*	LUC, GEN MOD	(90)
miR-150	Bone Marrow	No target(s) identified	N/A	(91)
miR-181	Bone Marrow	No target(s) identified	N/A	(92)
miR-221	Bone Marrow	*pten*	LUC, GE, 3UTR	(93)
miR-17~92	Germinal Center	**B Cells** *ikzf1* (Ikaros)	LUC, GE, GEN MOD	(94)
		**T Cells** *rora*	LUC, GEN MOD	(95)
		**T Cells** *pten*	LUC, GE, GEN MOD	(96)
		**T Cells** *bcl2l11* (Bim)	LUC, GE, GEN MOD	(96)
miR-21	Germinal Center	No target(s) identified	N/A	(97)
miR-28	Germinal Center	Many candidates	TA	(98)
miR-146	Germinal Center	**B Cells** *chuk* (IKKα)	LUC, GE	(99)
		**B Cells** *rel* (c-rel)	LUC, GE	(99)
		**T Cells** *icos*	LUC, GE, GEN MOD	(100)
miR-155	Germinal Center	**B Cells** *aicda* (AID)	LUC, 3UTR, GEN MOD	(101, 102)
		**B Cells** *socs1*	GE, GEN MOD	(101)
		**T Cells** *peli1*	LUC, CLIP, GEN MOD	(103)
miR-217	Germinal Center	Many candidates	TA	(104)
miR-155	Extrafollicular	*spi1* (PU.1)	LUC, 3UTR	(105, 106)
miR-182	Extrafollicular	No target(s) identified	N/A	(107)

LUC, luciferase assay or equivalent assay (e.g. GFP); GEN MOD, genetic modulation of target gene/rescue; GE, gene expression analysis in miRNA KO/overexpression; 3UTR, 3’ UTR binding site mutation; CLIP, CLIP assay; BP, binding Prediction; TA, transcriptomic analysis; Occurs in B cells or hematopoietic progenitors (in bone marrow) if not indicated.

### Regulation of Multiple miRNAs Is Involved in Early B Cell Development

Multiple steps, outlined earlier, are involved in B cell development. miRNAs have been shown to fluctuate in expression throughout the different stages of B cell development in the bone marrow, supporting the idea that their expression is important for guiding B cells through this process (108). Broadly, deletion of DGCR8 in B cells, which inhibits miRNA processing, caused a block in B cell development from the pro-B cell to pre-B cell stage (109). This was due to increased apoptosis of pro-B cells and resulted in a severe loss of B cells in the periphery of these mice (109), indicating that global miRNA expression is indispensable for B cell development.

**Figure 3:**
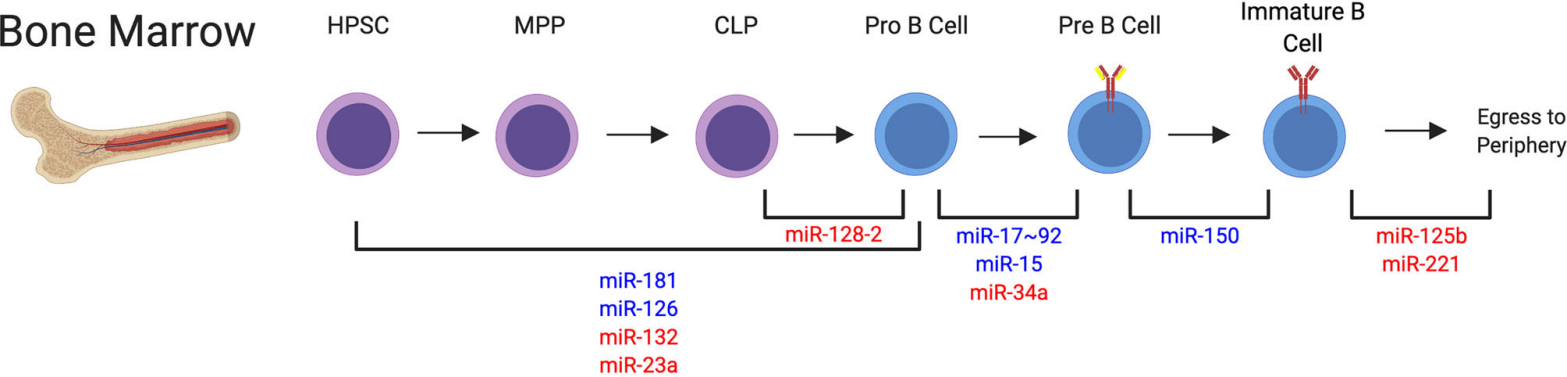
miRNAs that Impact B Cell Development in the Bone Marrow. Many miRNAs have been identified in the regulation of B cell development through each of the individual stages. This indicates a dynamic regulation of miRNA expression is required for proper developmental programs. miRNA regulation involved at these different stages is depicted. miRNAs that positively regulate these specific steps are depicted in blue. miRNAs that negatively regulate these specific steps are depicted in red. miRNAs are indicated at their confirmed or predicted stages of activity. HPSC, hematopoietic progenitor stem cell; MPP, multipotent progenitor; CLP, common lymphocyte progenitor.

Specifically, multiple studies have found important roles for several miRNAs during early fate decisions that polarize progenitors to the B cell lineage, or alternatively the T cell or myeloid cell lineages (**Figure 3**). Early expression of miR-181 or miR-126 in hematopoietic progenitor cells resulted in increased commitment to the B cell lineage (88, 92). miR-126 was shown to target IRS-1 to drive this commitment decision (88). Alternatively, expression of miR-132 or miR-23a in hematopoietic progenitors or miR-128-2 in common lymphoid progenitors (CLPs), resulted in reduced B cell lineage commitment, indicating that these miRNAs negatively regulate differentiation into the B cell lineage (85, 89, 90, 110). Mechanistically, miR-23a was able to regulate multiple transcription factors, including Ik2f1, Bach1, Satb1, and Runx1 (85), whereas miR-132 was shown to target Sox4 which was previously implicated in B cell development (90). Additionally, apoptosis modulation was responsible for a miR-128-2 dependent increase in CLPs, with A2B and Malt1 identified as candidate targets of miR-128-2 (89). The exact mechanisms of miRNA targeting involved in the activity of miR-181 in this process remains an open question.

Following lineage commitment, miR-17~92 was shown to increase PI3K activity in pro-B cells to regulate RAG expression and allow for transition to the pre-B cell stage (84). Further, miR-15 was shown to be involved in the induction of transcriptional programming required for the differentiation to the pre-B cell stage, with cyclin E1 identified a direct target gene of miR-15 and cyclin D3 identified as an indirect target of this miRNA (83). Additionally, differentiation from the pro to pre-B cell stage was revealed to be sensitive to the levels of miR-34a expression, which must be reduced to allow expression of Foxp1, a direct target of miR-34a, to occur (86).

Multiple studies also support the activity of miRNAs in later stages of B cell development in the bone marrow. miR-150 is likely involved in the transition from the pre-B cell to immature B cell stage, although aberrant premature expression can block development at earlier stages in the bone marrow (91). Downregulation of miR-125b and miR-221 have been shown to promote egress of B cells to the spleen, with S1PR1 and PI3K signaling regulation involved in this process (87, 93, 111). It is less clear which miRNAs are alternatively upregulated to promote B cell egress from the bone marrow. Ultimately, these studies indicate that there is a dynamic regulation of miRNA expression that controls the multiple stages of B cell development in the bone marrow, with a delicate balance of miRNAs providing both positive and negative regulation of these responses (**Figure 3**). Accordingly, improper miRNA expression can generate excessive B cell responses, with clinical manifestations of malignancy (112) or autoimmunity (to be discussed in detail). Mouse studies also suggest that miRNAs could play a role in clinical immunodeficiency syndromes such as severe combined immunodeficiency (SCID), since the discussed studies indicate that miRNA function is required for proper B cell development. However, the exact role of miRNAs in clinical immunodeficiency observed in human patients requires further study.

### Identifying and Determining the Requirement for miRNAs in GC Response

The contributions of miRNAs to the GC response have also been extensively documented. Similar to analyses assessing the overall importance of miRNAs in early stages of B cell development in the bone marrow, the loss of Dicer function (and thus the inability to generate mature miRNAs) in B cells undergoing class-switching *via* an AID-Cre based system effectively ablated the GC response and class-switched antibody production in mice (113). Likewise, the loss of DGCR8 function in T cells prevented the differentiation and function of follicular helper T cells (Tfh) and in turn GC B cells (95). These studies indicate that miRNA function is generally indispensable for the establishment of GC responses.

**Figure 4:**
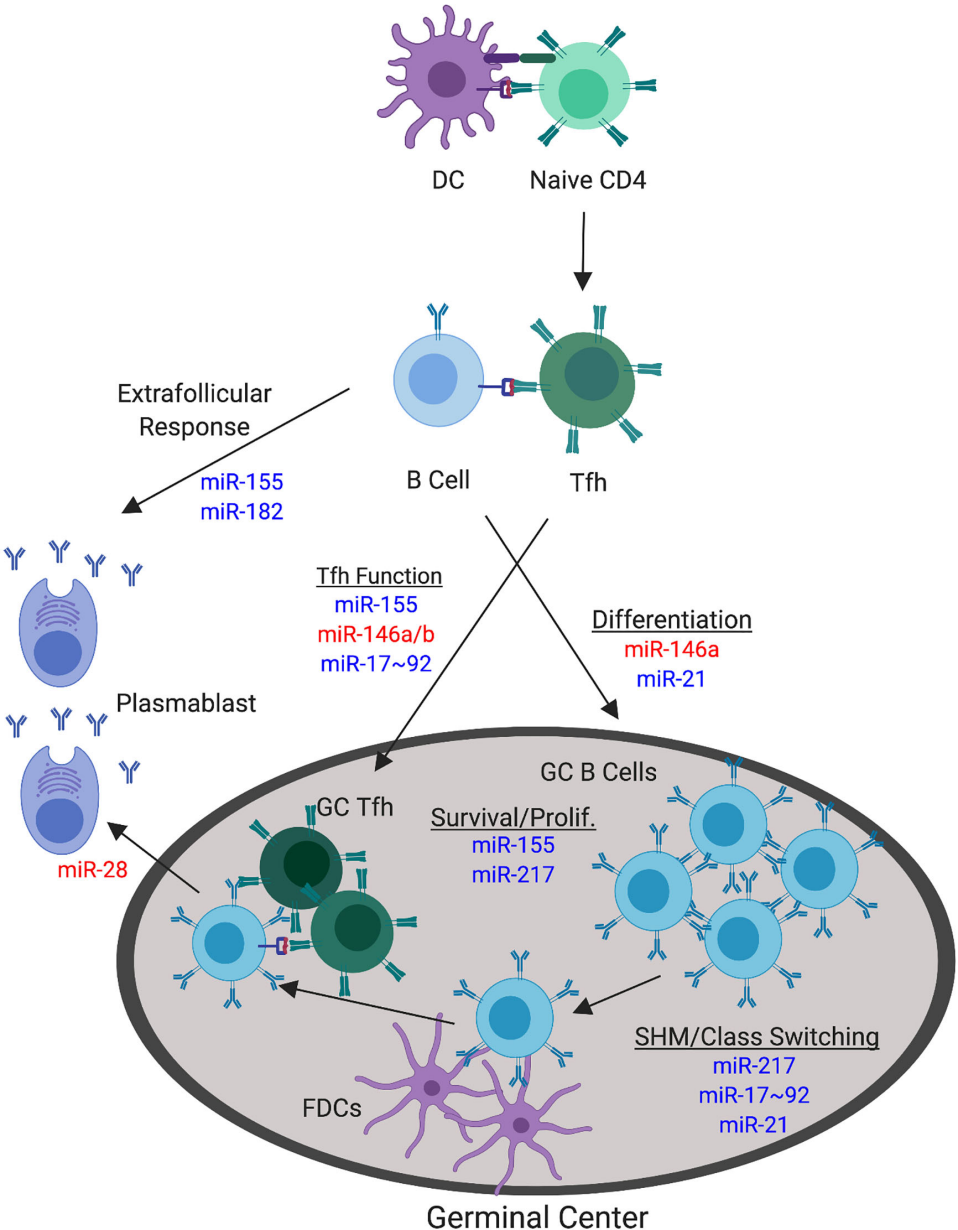
miRNAs with Confirmed Functions in the Non-autoimmune GC Response. The germinal center (GC) response involves a number of processes that can be targeted by miRNA function. The focus herein pertains to direct modulation of GC B cell and Tfh responses during normal, non-autoimmune GC responses. Major processes involved in the GC response are underlined. miRNAs that positively regulate these specific GC processes are depicted in blue. miRNAs that negatively regulate these specific GC processes are depicted in red. miRNAs are indicated at their confirmed or predicted stages of activity.

The hindrance of miRNA processing machinery represents a global loss of miRNA function. Additional studies that profile the expression of miRNAs in GC B cells and Tfh, as compared to their respective precursor cells, have identified specific miRNAs that may be absolutely crucial for mediating the differentiation and function of these specific cell types (100, 103, 114–116). We will further discuss those that have been studied in mouse models (**Figure 4**).

### miR-155 Is a Positive Master Regulator of the GC Response

miR-155 has emerged as arguably the most important and well-studied miRNA during the GC response, acting as a master regulator, with both B and T cell intrinsic functions demonstrated to date. The requirement for miR-155 in GC responses and subsequent antibody production was first described in 2007 through the implementation of both overexpression and knockout systems in mice (117, 118). The absence of miR-155 resulted in reduced GC responses in both the lymph nodes and Peyer’s patches, whereas overexpression enhanced these responses following immunization. A downstream effect on antigen-specific antibody production following immunization in this system was also observed (117). Subsequent studies of miR-155 function separated its B and T cell-intrinsic functions. B cell-intrinsic miR-155 expression was required for optimal GC responses and Ab production, with a primary effect on IgG1, following immunization (105). miR-155 was found to functionally target AID (*aicda*), as well as SOCS1 expression to promote cell survival *via* control of p53 (101, 102). The targeting of AID is somewhat counterintuitive as AID is required for somatic hypermutation and class-switching. However, despite AID being a verified target of miR-155, miR-155 deficiency did not overtly affect somatic hypermutation or class-switching processes when measured directly (102, 117). Therefore, the deficiency in class-switched antibody production in the absence of miR-155 is more likely associated with reduced differentiation and the survival of plasmablasts, which is further discussed later in regard to the effects of miR-155 on the extrafollicular B cell response (119). In addition to B cell-intrinsic effects of miR-155, miR-155 deficiency in T cells resulted in significantly blunted Tfh and GC B cell responses, as well as primary and memory antibody responses, demonstrating non-redundant roles for miR-155 in B and T cells that lead to similar phenotypic effects (103, 120). Mechanistically, miR-155 was found to target *peli1* in T cells to increase c-Rel expression during Tfh development, resulting phenotypically in the modulation of proliferation and CD40L expression (103). Collectively, miR-155 targets an array of genes and processes in B and T cells during the formation and activity of the GC response and can be considered a master regulatory miRNA during this process.

### The miR-146 Family Negatively Regulates the GC Response

In addition to the well-described and multifaceted function of miR-155 in promoting GC responses, both miR-146a and miR-146b have emerged as negative regulators of the GC response. Further, miR-146 modulation also occurs within both B and T cells. Carola Vinuesa’s group first showed that miR-146a loss in T cells through a mixed bone marrow chimeric approach resulted in the spontaneous expansion of Tfh and GC B cells, with some added effect of T cell extrinsic factors (100). As modulation of ICOS-ICOSL signaling through blockade or genetic methods could rescue Tfh and GC B cell accumulation, miR-146a modulation of this signaling axis was determined as a significant form of action utilized to spontaneously control Tfh numbers (100). Another study later clarified that B cell-intrinsic miR-146a deficiency following immunization-induced response does indeed result in increased GC B cell, Tfh, and antibody responses in part due to control of CD40 signaling and control of the GC B cell differentiation process (99). However, this study did not find an effect of miR-146a alone on the modulation of Tfh responses both spontaneously and following immunization when using a Cre-flox system (99), exhibiting contrasting results to previous study. Instead, they found a cooperative T cell-intrinsic role of miR-146a and miR-146b in this process (99). While these results are slightly divergent, it is clear that the miR-146 family is collectively responsible for the negative regulation of the GC, demonstrating that miRNAs can both positively and negatively regulate GC responses.

### The miR-17~92 Cluster Modulates Tfh Responses

While miR-146 is critical in negative regulation of Tfh responses, the miR-17~92 cluster has conversely emerged as a critical T cell-intrinsic positive regulator of Tfh, GC, and antibody responses as detailed by multiple studies employing immunization, viral infection, and spontaneous systems in non-autoimmune mice (95, 96, 121, 122). PTEN and Bim (*bcl2l11*) were the first identified targets of the miR-17~92 cluster in CD4 T cells and loss of one allele each of *Pten* and Bim (*bcl2l11*) could produce a similar phenotype to the overexpression of miR-17~92 (96). More convincingly, the loss of one copy of *Pten* in mice lacking miR-17~92 in T cells could rescue the response, further suggesting that PI3K signaling is critical for miR-17~92 function (121). In addition to the promotion of Tfh function, the activity of miR-17~92 inhibits the expression of factors associated with other CD4 T cell subsets, such as the direct target of *rora* (95). A separate study tested the B cell-intrinsic requirement for miR-17~92 and did not observe a difference in GC formation, though miR-17~92 had a drastic effect specifically on the production of IgG2c (94). These data suggest that miR-17~92 primarily acts in a T cell-intrinsic manner to modulate GC responses through promoting Tfh differentiation. Conversely, its B cell intrinsic functions appear less pertinent, or may only be involved in specific types of responses.

### Other miRNAs Explored in the GC Response

While the study of miR-155, miR-146, and miR-17~92 has been of primary focus in the GC field, additional studies utilizing mouse models are emerging to both support and exclude the role of other miRNAs in this process. In one study, the overexpression of miR-217 in mice resulted in enhanced GC B cell and antibody responses, including enhanced somatic hypermutation events, during primary and secondary responses following immunization (104). Conversely, dampening miR-217 function resulted in reduced GC B cell and antibody responses, indicating that miR-217 promotes these events (104). These responses were associated with the prevention of Bcl6 degradation in GC B cells (104). In a separate study, miR-28 modulation did not affect the magnitude of the GC B cell response, but the employment of a miR-28 sponge in transferred B cells resulted in enhanced memory formation and plasma cell differentiation, indicating a cell-intrinsic negative regulation during GC B cell terminal differentiation into plasmablasts (98). Lastly, we identified a role for miR-21 in driving GC responses to foreign antigen. miR-21 deficient mice exhibited a two-fold reduction in the magnitude of the GC response and reduced class-switched IgG antibody responses after immunization, indicating that miR-21 is required for optimal GC response to foreign antigen (97).

Additionally, some miRNAs are highly expressed in GC B cells or Tfh, but are dispensable for *in vivo* responses. Among these miRNAs are those contained in the miR-183 cluster (miR-182, miR-183, and miR-92) which are upregulated in GC B cells and Tfh (103, 123, 124), as well as miR-22 which is specifically upregulated in Tfh (103). However, it is worth noting that miRNA requirement may be specific to the type of ongoing B cell and GC response, so these results may not hold true for all conditions. miRNAs that have similar seed sequences may also exhibit some redundancy, meaning that loss of both miRNAs may be required to observe a phenotype *in vivo*. In summary, the miRNAs that contribute at different stages of the GC response are depicted in **Figure 4**.

### miR-182 and miR-155 Are Implicated in Extrafollicular B Cell Responses

Overall, very little is currently known about the miRNAs involved in any form of extrafollicular B cell response, beyond a role for miR-182 and miR-155 (105, 107). miR-182KO mice immunized with T-dependent antigen (TD-Ag) exhibited intact GC responses, but showed reduced antibody responses at early time points following immunization, indicating an impairment in early extrafollicular B cell responses (107). This reduced early response did not affect the ability to elicit memory responses (107). Similar to miR-182KO mice, B cell intrinsic deficiency of miR-155 resulted in reduced antibody forming cell (AFC) responses at 7d post-immunization with TD-Ag, indicating impaired extrafollicular responses (105). This was shown to be dependent on miR-155 mediated regulation of PU.1 expression, which regulates the formation of plasmablasts (105, 106). Study of miR-155 indicates that some miRNA function in B cells can impact both the GC and extrafollicular B cell responses, likely because there is much crosstalk in the factors that must be activated to support both of these processes. It is likely that additional miRNAs are involved in extrafollicular B cell responses against T-dependent foreign antigens, but further study is required to identify these factors.

### Considerations for Future Study of miRNAs in Protective B Cell Responses

While multiple miRNA profiling studies have been performed on B cells undergoing developmental processes and GC responses, these profiling studies are lacking to identify miRNAs which may be involved during the extrafollicular B cell response. In general, many mechanistic questions remain in regard to extrafollicular B cell function and identifying critical miRNAs and their targets can help speed up discovery of additional important pathways that mediate this response. Further, while many studies have illustrated that modulated expression of multiple B cell intrinsic miRNAs is crucial for B cell development in the bone marrow, miRNA expression in other cell types during this process such as stromal cells may also significantly impact B cell development but remain unexplored. Similarly, in addition to the B cell and T cell-intrinsic effects of miRNAs on GC responses, miRNA expression in other cell types involved in the establishment of GC responses, (i.e. follicular dendritic cells (FDCs)), Tfh-priming DCs, and T follicular regulatory cells) may significantly affect their optimal formation. Some studies have started to profile miRNA expression using FDC and FDC-like cell lines (125, 126), however further study is required to determine if this accurately represents what is observed *in vivo*. Additionally, many miRNAs modulate the cytokine microenvironment, providing a potential indirect mechanism of GC control.

The availability of mouse models and reagents for miRNAs of interest is critical in the continued study of their contribution to this process. Additionally, most of the targets identified thus far were discovered using immunization-based systems, meaning that it is unclear which miRNAs are similarly or differentially modulated in spontaneous GC and B cell responses in the periphery. Some studies have started to address this and will be mentioned in the next section. Notably, some miRNAs such as miR-17~92 and miR-155 appear to be involved throughout multiple stages of B cell development and are critically tied to many important B cell fate decisions. Thus far, other miRNAs have only been described at one stage of B cell response. Establishing a complete picture of miRNA responses at all stages of the B cell response is an important consideration in regard to any therapeutic pursuits for autoimmune or other B cell dependent diseases.

## miRNA Regulation of B Cell Responses in SLE

**Figure 5:**
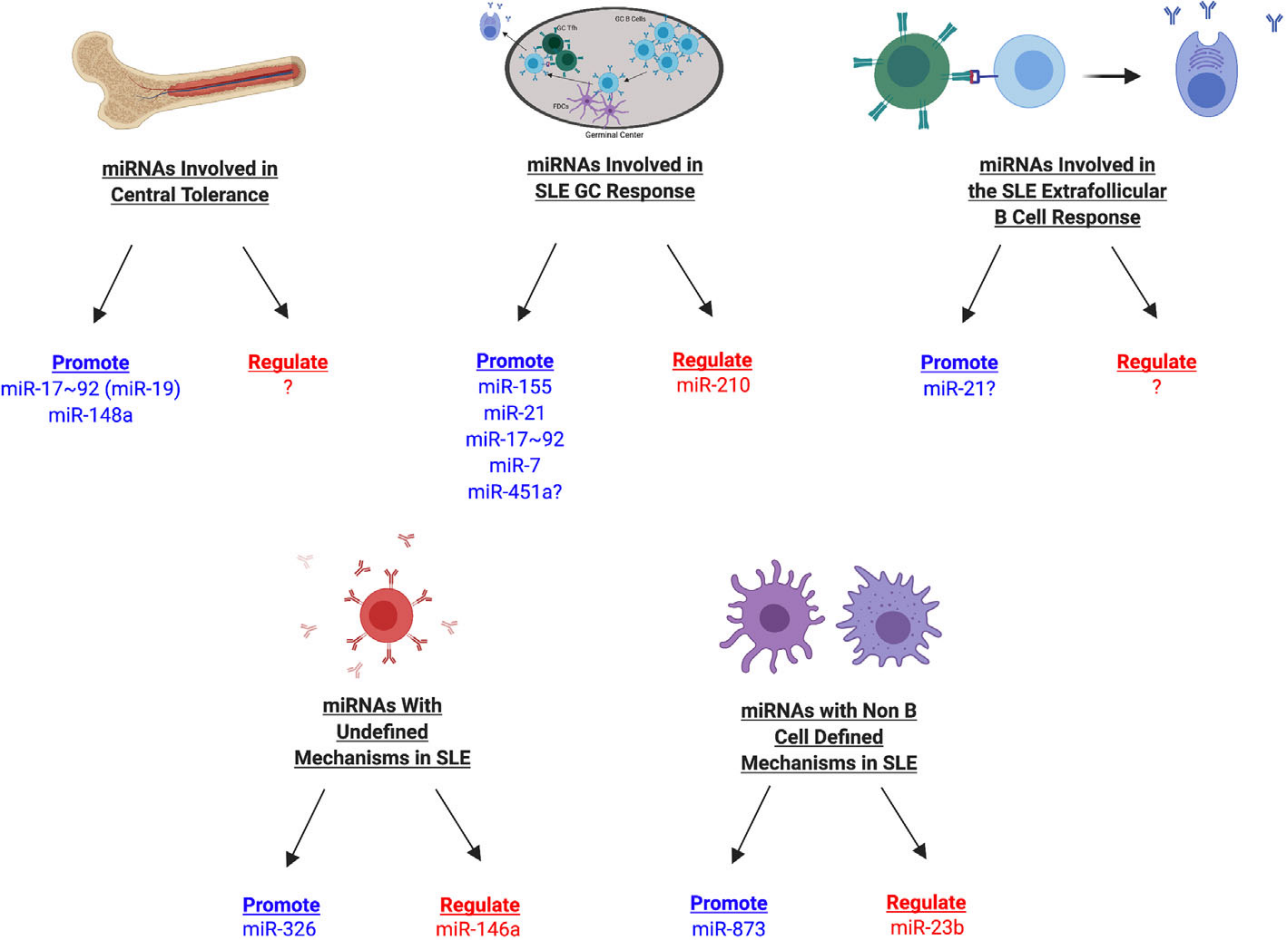
miRNAs Involved in SLE Development in Mice. miRNAs involved in SLE development can be divided into multiple categories depending on their association with different stages of the B cell response (central tolerance, germinal center, extrafollicular response, other mechanisms), in addition to whether they promote or regulate disease manifestations. Those miRNAs that promote disease are shown in blue whereas those that regulate disease are shown in red. miRNAs with currently undefined mechanisms in SLE may later be identified during a specific stage of the B cell response or may have B cell independent mechanisms during SLE.

Due to the critical involvement of miRNAs at many stages of the B cell response, it is not surprising that dysregulated miRNA expression can lead to the loss of B cell tolerance at multiple stages of B cell development and effector function. As such, global miRNA expression in B cells, studied with a B cell specific knockout of Dicer, was shown to skew the B cell repertoire and result in high titers of serum autoreactive antibodies and kidney pathology (127). This study illustrated that miRNAs can collectively impact B cell tolerance. We will further discuss which individual miRNAs have been characterized in this process and what is known about the mechanisms of their activity (**Figure 5** and **Table 3**).

**Table 3:** miRNAs and Direct Target Genes in SLE Responses.

miRNA	Site	Confirmed or Predicted Target Gene(s)	Key Assays Performed	Ref #
miR-17~92	Bone Marrow	*pten*	LUC, GE, GEN MOD	(128)
miR-148a	Bone Marrow	*bcl2l11* (Bim)	LUC, GE, GEN MOD	(129)
		*pten*	LUC, GE, GEN MOD	(129)
		*gadd45a* (Gadd45α)	LUC, GE, GEN MOD	(129)
miR-7	Germinal Center	*pten*	GE, LUC	(130, 131)
miR-17~92	Germinal Center	**Lymphocytes** *pten*	LUC, GE, GEN MOD	(96)
		**Lymphocytes** *bcl2l11*(Bim)	LUC, GE, GEN MOD	(96)
miR-21	Germinal Center	Many candidate target genes	TA	(97)
miR-145a	Germinal Center	**Whole Spleen** *irf8*	LUC, GE	(132)
miR-155	Germinal Center	**Whole Spleen** *s1pr1*	TA, LUC, GEN MOD	(133)
		*inpp5d* (SHIP-1)	GE	(134)
miR-210	Germinal Center	*cd23* (but likely additional targets)	LUC, TA	(135)
miR-23b	Kidney	**Non-immune cells** *tab2*	TA, LUC, GE, GEN MOD	(136)
		**Non-immune cells** *tab3*	TA, LUC, GE, GEN MOD	(136)
		**Non-immune cells** *chuk* (IKKα)	TA, LUC, GE, GEN MOD	(136)
miR-146a	Spleen	**Global** *traf6*	GEN MOD	(137)
miR-155	Lung	**Lung tissue** *ppara* (PPARα)	GE, TA, LUC, CLIP	(138)
miR-326	Plasmablast (GC/EF)?	*ets1*	GE	(139)
miR-873	Spleen	**T Cells** *foxo1*	LUC, GE	(140)

LUC, luciferase assay or equivalent assay (e.g. GFP); GEN MOD, genetic modulation of target gene/rescue; GE, gene gene expression analysis in miRNA KO/overexpression; CLIP, CLIP assay; TA, transcriptomic analysis; Occurs in B cells or hematopoietic progenitors in bone marrow if not indicated.

### miR-17~92 and miR-148a Break Central B Cell Tolerance

Several miRNAs have been identified as key mediators of central B cell tolerance. The implementation of the IgM^b^-macroself mouse model has recently been adopted as a mechanism to study central tolerance (141). IgM^b^-macroself mice express an IgM^b^ superantigen and editing of the BCR in the bone marrow is unable to remedy this reactivity, resulting in deletion of nearly all B cells in the bone marrow and consequent loss of B cells in the periphery. Therefore, this model allows for the assessment of B cell tolerance by screening for B cells that have escaped this method of tolerance and entered the periphery. In one study utilizing this model, IgM^b^-macroself mice reconstituted with bone marrow from CD19-Cre; miR-17~92 mice revealed that overexpression of this miRNA cluster allows developing B cells to escape central tolerance in the bone marrow (128). Most of this effect was attributed specifically to the miR-19 subfamily suppressing Pten activity (128). Further, normal levels of miR-17~92 were shown to regulate the degree of receptor editing occurring at this stage of tolerance, indicating that some level of miR-17~92 expression is important to mediate normal processes that occur during B cell development (128).

A separate study performed a functional screen of 113 miRNAs separated into 4 pools in the regulation of central B cell tolerance, also employing the IgM^b^-macroself mouse model. From this screen, they identified 7 miRNAs that may be involved in escape of central tolerance. Using this model to study the effect of specific miRNAs, they determined that miR-26a, miR-26b, miR-342, miR-423, and miR-182 have modest effects on central tolerance, whereas miR-148a was identified as a key regulator of central tolerance, with increased levels promoting escape of tolerance (129). Mechanistically, miR-148a was found to regulate immature B cell apoptosis through targeting Bim, PTEN, and Gadd45α, which were also verified to regulate central tolerance in this mouse model (129). Consequently, MRL/lpr mice overexpressing miR-148a developed accelerated autoimmune manifestations and higher autoantibody titers (129).

While these studies have made significant progress toward our understanding of miRNAs that regulate central B cell tolerance, some questions still remain. Gonzalez-Martin et al. screened a subset of 113 miRNAs in hematopoietic progenitor stem cells (HPSCs). Downstream determination of miRNAs involved in loss of central B cell tolerance was dependent on their enhanced expression in B cells that seeded the spleen in their system. However, expression of these miRNAs in other cell types may indirectly impact B cell escape of central tolerance. Additionally, other miRNAs that were not screened in this study may have a significant contribution to this process. Once B cells are in the spleen, they are also regulated at the transitional stage. Little is currently known about the involvement of miRNAs in transitional B cell tolerance, but miRNAs have been identified in regulating BCR signaling induced growth and apoptosis, indicating that these may be prime candidates for the study of tolerance at the transitional stage of B cell development (142).

### miRNAs in SLE-Associated GC Responses

Based on the master regulatory function of miR-155 in the GC responses, it is not surprising that miR-155 has a great importance in the onset of SLE in two different mouse models. Fas^lpr^ mice with a deficiency in miR-155 have reduced spleen size, proteinuria, and kidney pathology with age (133, 134). This was associated with reduced serum autoantibody titers and GC responses in this model (133, 134), illustrating that miR-155 is also important in promoting the formation of GCs in autoimmune-prone mice. In one of these studies, miR-155 was shown to target expression of SHIP-1 (*inpp5d*), a previously characterized miR-155 target gene (143), following BCR activation to promote B cell proliferation (134). In the other study, S1PR1 was validated as a miR-155 target. Antagonizing S1PR1 expression lead to increased Tfh responses (133), revealing another mechanism of miR-155 activity. In the pristane-induced lupus model, knockout or antagonism of miR-155 dampened diffuse alveolar hemorrhage, reduced kidney pathology, and reduced autoantibody titers, demonstrating common effects among multiple lupus models (138, 144).

Another miRNA which has been studied using multiple SLE models is miR-21. Antagonism of miR-21 was initially shown to dampen splenomegaly and autoantibody titers in the Sle1.2.3 lupus model although no detailed mechanism of action was suggested in this seminal study (145). This reduction in splenomegaly and autoantibody production was also observed in the bm12 adoptive transfer chronic graft versus host disease (cGVHD) model, which exhibits SLE-like manifestations (146). We recently addressed more concerning the mechanism of miR-21 activity and showed that miR-21 has context dependent effects in different SLE models, which is likely related to the level of miR-21 activity (97). In a TLR7 induced model where *Sle1b* mice are treated with imiquimod, miR-21 modulated plasma cell formation and autoreactive B cell selection, which was independent of the magnitude of the GC response and was associated with the modulation of a number of miR-21 target genes in B cells. In addition to differences in the B cell response, myeloid cell infiltration and proinflammatory cytokine production was blunted in the absence of miR-21, suggesting that miR-21 can also control the cytokine environment (97). In contrast, in a spontaneous TLR7 overexpression model, *Sle1b*.Yaa, miR-21 was required for increased GC responses and autoimmune B cell responses (97). These data indicate that miR-21 has multifaceted effects at several different stages of the B cell response depending on the SLE model. Additional study is required to determine the cell-intrinsic contributions to these distinct responses.

While the impact of miR-17~92 on autoimmunity has not yet been tested in a standard mouse model of SLE, preliminary - study indicates that there will likely be broad effects among several SLE models. Overexpression of miR-17~92 in lymphocytes results in multiorgan autoimmune manifestations, including lymphoproliferation, kidney pathology, and the development of autoantibody titers (96), hallmarks of SLE. These mice exhibited increased GC responses (96), but the study of central tolerance suggests that miR-17~92 can affect tolerance at multiple stages of B cell development and effector function (128).

In addition to these miRNAs, multiple other studies have illustrated roles for additional miRNAs in GC responses and autoimmunity. miR-7 was studied in the MRL/lpr mouse model and was found to enhance GC responses, drive proinflammatory cytokine secretion, and impact autoantibody production and kidney pathology (130). Treatment of MRL/lpr mice with antagomir-7 for 5 weeks was able to reduce all of these responses significantly and normalized PTEN expression in B cells (130, 131). Another study addressed miR-451a deficiency in the Fas^lpr^ model and found a partial reduction in Tfh responses and a significant reduction in IL-21 production that correlated with the loss of autoantibody production and kidney pathology (132). IRF8 was verified as a gene target (132). While IRF8 is important in the GC response (147), it is unclear to what extent IRF8 modulation produces the observed effects and that what extent other mechanisms may be at play. Lastly, another study hinted that miR-210 may be involved in regulating the loss of tolerance through the GC, as the loss of miR-210 resulted in spontaneous development of autoimmunity and enhanced spontaneous GC B cell responses with age (135). The exact mechanisms of miR-451a and miR-210 in the GC responses in autoimmune-prone mice remain to be fully elucidated.

### miRNAs That Modulate B Cell Tolerance *via* Other or Undefined Mechanisms

Another well studied miRNA in autoimmunity is miR-146a, which was found to prevent the development of autoimmunity, similar to its regulatory effects on the GC responses to TD-antigen immunization or spontaneous responses in non-autoimmune mouse models. This was determined by two studies employing opposite approaches. First, in the more classical BXSB model, administration of miR-146a expressing virus like particles (VLPs) reduced autoantibody production (148). Second, even in the absence of susceptibility loci, miR-146aKO mice develop a chronic inflammatory disease and autoantibody production with age (149). While it is evident that non-autoimmune spontaneous-GC responses are affected in miR-146aKO mice through a separate study previously discussed (100), analysis in autoimmune-prone mice did not directly address this mechanism and thus further study of GC responses in autoimmune-prone mice is required to determine if alteration of the GC response may be a causative factor under these conditions. However, it is difficult to directly attribute autoimmunity solely to lymphocyte dysregulation in the absence of miR-146, as these mice also exhibit a profound amount of myeloproliferation when miR-146a is globally deficient, which can be significantly reduced by attenuation of the miR-146a target gene TRAF6 (137, 149). It is likely that a combination of these factors may be involved in contributing to the overall phenotype observed, which requires further study. Additional study of human SLE genetics has revealed that the rs2431697 SNP is critically tied to miR-146a expression levels and the T/T variant at this locus correlates with disease activity, renal involvement, and autoantibody production (150). This is linked to its alterations in cell type specific enhancer activity that can control miR-146a expression by promoter interactions (151). These studies indicate that miR-146a activity has relevance in human SLE and that genetics can control miR-146a expression in SLE patients.

miR-326 is also intriguing due to its modulation of B cell responses. Enhancing miR-326 expression *via* lentiviral vector in MRL/lpr mice resulted in enhanced autoantibody production and immune complex deposition in the kidneys, which was associated with increased plasmablast responses (139). Ets-1 expression was modulated in B cells, with Ets-1 previously identified as a miR-326 target gene (152). However, the source of plasmablast generation (GC versus extrafollicular pathway) was not characterized and reverse experiments implementing lentivirus expressing miR-326 sponge showed only minimal effects on these processes, implying the need for further inquiry (139).

Two additional miRNAs have detailed roles in the development of autoimmunity but are associated with other divergent mechanisms focused on T cell and cytokine responses. miR-873 inhibition could modestly reduce autoantibody responses and proteinuria in MRL/lpr mice, but was associated with Foxo1 targeting and Th17 responses (140). MRL/lpr mice infected with a viral vector expressing a miR-23b sponge or virus engineered to overexpress miR-23b demonstrated that miR-23b has a suppressive effect on the development of kidney pathology (136). This study attributed miR-23b mediated protection from autoimmunity to its negative regulation of proinflammatory cytokine production in resident non-immune cells (136).

### Considerations for Further Study

Altogether, miRNAs crucially regulate autoimmune responses, in both negative and positive manners (**Figure 5**). While these miRNAs have already been established in this context, much study remains to identify additional miRNAs involved in autoimmunity, as well as to further determine the mechanisms involved in these processes. The list of miRNAs that modulate autoimmunity through regulation of the B cell responses is likely to grow as the tools and reagents to study these factors expand. For example, recently miR-152-3p was identified as an important miRNA in isolated human SLE B cells, which controls their activation level and production of BAFF (153). Modeling this miRNA in mouse models may reveal more information about the importance of this miRNA in driving disease progression.

Interestingly, none of the miRNAs listed above are located on the X chromosome (154). However, it would be interesting to determine if aberrant miRNA expression caused by incomplete X chromosome inactivation may contribute to the pathogenesis of SLE. Additionally, the study of miR-146a indicates that genetic susceptibility to SLE may involve mutations that lead to aberrant regulation of miRNA expression that predisposes one to SLE development (150, 151). Additional studies addressing this potential phenomenon in regard to the expression of other miRNAs will be informative.

Of further consideration for future study is the level of miRNA expression that is required to promote or restrain disease, as many of these miRNAs are expressed during normal B cell processes, but at different levels. Many of these models focus on extreme alterations in expression, either by knocking out expression entirely or significantly overexpressing the miRNA by viral vector. Approaches that fine-tune miRNA expression, such as using heterozygous mice or miRNA sponges, should be considered in future studies.

**Figure 6:**
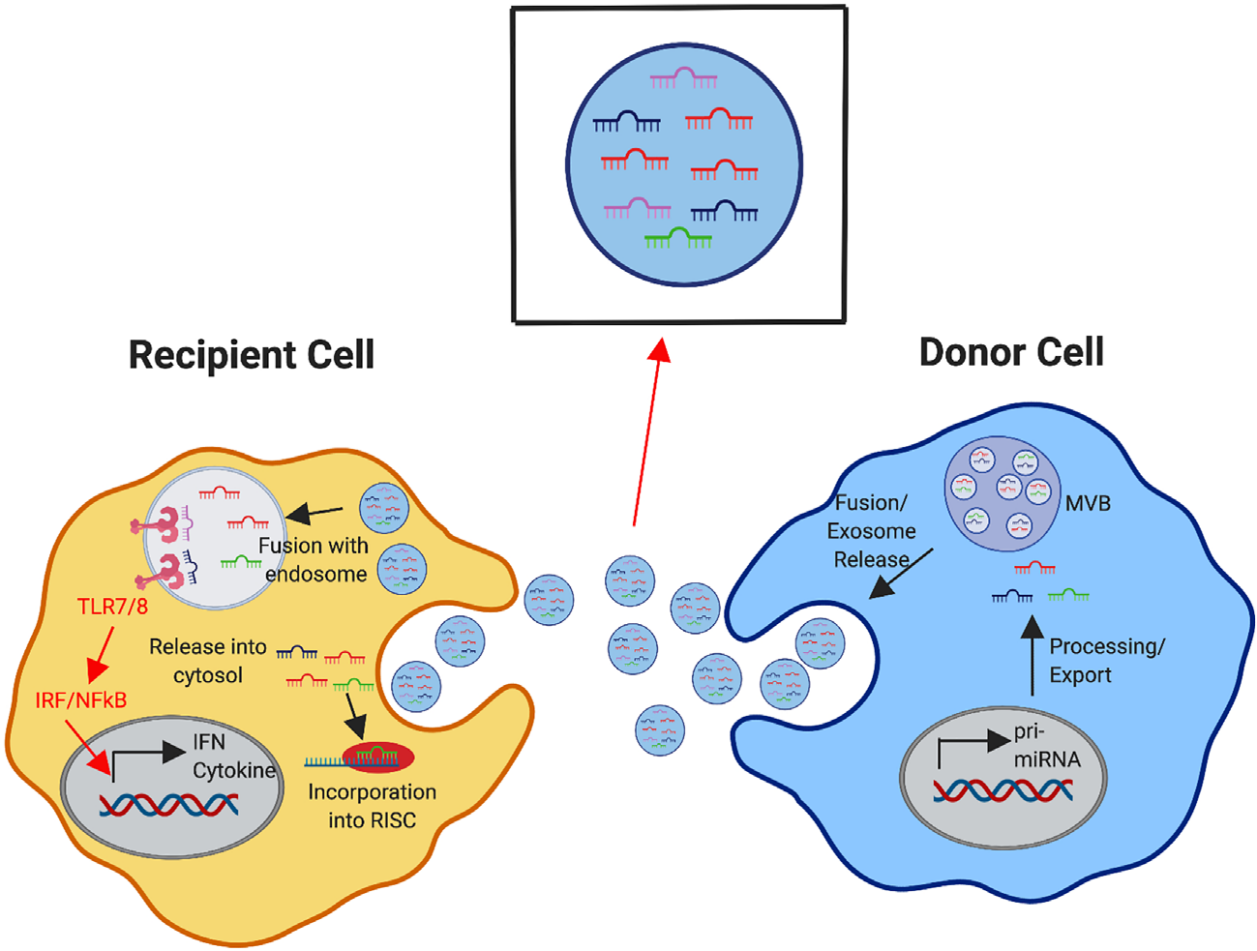
Exosomal Delivery of miRNA to Modulate Recipient Cell Response. Donor immune cells produce and process miRNAs (as depicted in detail in **Figure 1**). The formation of multivesicular bodies (MVBs) with intraluminal vesicles containing mature miRNAs in the donor cell leads to fusion of the MVBs with the plasma membrane, releasing exosomes containing these miRNAs. Recipient immune cells can receive exosomal cargo into the cytosol by direct fusion of the exosome with the plasma membrane or exosomes can be internalized and delivered to the endosomal compartment. Within the cytosol, these received miRNAs may compete with the internally generated miRNAs for incorporation in the RNA-induced silencing complex (RISC) to mediate gene expression in the recipient cell. miRNAs that are directed into the endosomal compartment may bind to TLR7/8 to initiate downstream signaling cascades, triggering the induction of cytokines, interferons, and other interferon stimulated genes.

While the experimental focus on miRNA has largely pertained to their classical mechanisms of function, other mechanisms of miRNA activity are emerging that require consideration and may alter the manner in which we think about targeting them. There is evidence that miRNAs can also act as ligands by binding to and inducing pattern recognition receptor (PRR) signaling, predominately by TLR7 and TLR8, which recognize ssRNA ligands (155–157). The idea of miRNAs being able to stimulate TLRs is intriguing as this opens the door for miRNAs produced by one cell to control the genetic profile and function of surrounding cells. In fact, it has already been shown that T cells can transfer exosomes and their miRNA cargo to APCs through the immunological synapse to mediate fusion and release into the recipient cell (158). Additionally, DCs can shuttle miRNAs *via* exosomes to the cytosol of other DCs to mediate recipient gene expression (159). Interestingly, exosomes derived from SLE patients can stimulate the production of IFNα from pDCs *in vitro* through miRNA stimulation of TLRs, indicating that exosomal delivery of miRNAs can also alter the cytokine profile in disease (160). Realistically, this means that the overall phenotype observed for specific miRNAs may actually be a balance between miRNAs produced intrinsically and those internalized from surrounding cells, which may result in TLR stimulation as well as gene regulation in the recipient cell (**Figure 6**).

An initial study recently applied this concept to the T and B cell interactions required for B cell class-switching and GC formation. This study found that during *in vitro* culture, T cells can transfer multiple miRNAs, including miR-155, *via* exosomes to B cells upon formation of immune synapses. Blocking exosome release from T cells to B cells *in vivo*, and thus reducing miRNA transfer from T cells to B cells, resulted in slightly reduced B cell class-switching and GC formation. This indicates that miRNA transfer contributes to these B cell responses *in vivo*, with greater contribution from intrinsic miRNA responses (161). It is unclear if this mechanism may be amplified during autoimmune responses and which cell types may participate in this mechanism in a context dependent manner.

Lastly, while miRNAs target individual genes in the cell, they have been implicated in targeting the chromatin remodeling machinery to confer epigenetic and transcriptional changes in the genome that have broader effects on cellular function. This includes targeting factors involved in methylation, acetylation, and the SWI/SNF complex (162–168). A few studies have started to address miRNA targeting of DNA methyltransferases (DMNTs) in CD4 T cells in SLE (19, 169, 170). However, the study of miRNAs in the modulation of the chromatin remodeling machinery has not been pursued extensively. This concept is intriguing though, because SLE has been associated with epigenetic modulation, particularly hypomethylation (171). Altogether, miRNA function may not be limited to a single mode of action, but rather a combination of effects that alter the functionality of a cell and surrounding cells. This has broad implications for the mechanistic control of both non-autoimmune and autoimmune responses.

## Considerations for miRNAs as Therapeutic Targets

miRNAs are an attractive therapeutic option for SLE and other diseases because of the ability to dampen their expression rather than completely ablate it, allowing for the ability to fine-tune gene expression to a desired level. In the context of SLE, this is an interesting concept because current therapeutics globally target B cells or employ other approaches that result in broad immunosuppression, thus ablating both anti-pathogen and autoimmune responses (172). In turn, this leaves patients on these therapies susceptible to infection. If miRNA expression can be fine-tuned to a level that allows for adequate anti-pathogen response, while significantly dampening autoimmune manifestations, miRNA targeting would be effective. Alternatively, finding miRNAs that promote autoimmunity but are not overtly involved in anti-pathogen response provides another attractive avenue of attaining specificity.

### Methods for Targeting miRNAs and miRNAs in Clinical Trials for Other Diseases

In regard to the specific methods of miRNA targeting, many groups have already worked with miRNA targeting in cell culture and in animal models through the introduction of antagomirs, which are 23 nucleotide antisense 2’-O’-methyl-modified oligonucleotide sequences bound to cholesterol (173) (**Table 1**). The cholesterol linkage allows for cellular entry while the antisense sequence promotes pairing to and neutralization of the target miRNA. Another form of anti-miRNA oligonucleotide is the locked nucleic acid (LNA) anti-miR agent. LNA anti-miRs are another form of targeted oligoribonucleotide that are stabilized due to locked nucleic acids (LNAs) (174). More recently LNA anti-miRs have been designed that are as short as 8 mer sequences (tiny LNAs) that specifically target the 5’ seed region of the miRNA (175). Of note, while mature miRNA can be targeted, the pri-miRNA and pre-miRNA stages of miRNA processing can be targeted by miRNA therapies as well, preventing generation of the mature miRNA (176). While anti-miRNA oligonucleotides are intended to dampen miRNA function, some miRNAs (i.e. miR-146a) have a regulatory role in disease onset and progression. Bearing this in mind, a separate approach is to heighten the expression of these miRNAs to restore tolerance, such as through the administration of a miRNA mimic (or agomir), which is a chemically modified double stranded oligonucleotide sequence designed to mimic the miRNA of interest.

While simple injection based approaches are limited to systemic effects, targeted delivery may be achieved through viral vectors utilizing different expression promoters (177). Also advantageously, viral vector based delivery methods can promote long-term modulation of the response through forming episomes or integrating into the genome (177). Anti-miRNA sequences expressed under the control of a viral vector are referred to as miRNA sponges (178). Alternatively, viral vectors can be engineered to express miRNAs. Lastly, nanoparticles are an attractive cell-specific targeting option that continue to be studied and optimized for a variety of purposes, including as small RNA delivery vehicles (179).

Despite miRNA therapeutics being a relatively newer pursuit, some miRNA-centric therapeutics are being tested in clinical trials for the treatment of diseases. The first miRNA-centric therapeutic to advance to Phase II clinical trials was Miraversin, which dampens miR-122 expression during hepatitis C infection to prevent viral replication (176, 180). As such, many companies have emerged with the goal of designing miRNA-based therapeutics for a variety of purposes (181). miRagen has already directed a miR-155 targeting oligonucleotide (Cobomarsen) into a Phase II clinical trial in an effort to treat blood cancer (182, 183). Additionally, therapeutics targeting other miRNAs, such as miR-21, miR-29, and miR-92, have completed or are currently active for early stage trials in the treatment of fibrotic diseases, cardiovascular disease, kidney disease, and solid organ cancers (181, 184). While the implementation of miRNA-centric therapeutics is steadily increasing, to our knowledge there are no trials to date that have focused on testing miRNA targeted therapeutics for autoimmune diseases. However, the technology exists for potential implementation.

### Considerations for Implementing miRNA Therapeutics in SLE

While miRNA targeting is an interesting therapeutic option, there are some points of consideration pertaining to off-target effects and the potential stimulatory ability of miRNAs, the necessity for long-term intervention in autoimmune diseases, and miRNA redundancy. Other points of consideration will likely emerge as more groups begin testing miRNA targeting in animal models. While antagomirs/anti-miRs are, by nature, targeted therapeutics due to their base-pairing properties, design must be careful and specific to avoid off target RNA binding. Despite this concern, study suggests that off-target effects on mRNAs are minimal when using LNAs in animal models (175). In addition to the generation of sequence specific off target effects, there is evidence that miRNAs can also act as ligands by binding to and inducing PRR signaling, predominately by TLR7 and TLR8, which recognize ssRNA ligands (155–157). If miRNAs can stimulate some PRRs, we must exercise caution in therapeutically enhancing miRNA doses that could trigger downstream proinflammatory cytokine production. Conversely, some antagomirs have been shown to block TLR7 and 8 signaling in a sequence specific manner in addition to their miRNA binding effects, possibly introducing other unintended effects (185). Careful modeling, design, and testing are required to overcome these barriers to specific therapeutic design.

Another point in regard to therapeutic development is how often antagomirs would need to be introduced into the system, as miRNAs are constantly transcribed from the genome in response to immune stimuli and SLE is chronic disease. Removal of the miRNA targeting therapy will likely result in a relapse of symptoms as the original expression levels are restored following therapy discontinuation. As discussed above, the use of viral vectors that mediate long-term expression is an option but requires further study. In addition to the frequency of miRNA dosage, it is unclear which miRNAs can be targeted therapeutically or only prophylactically in regard to SLE and other autoimmune diseases. Ultimately, this likely depends on the specific miRNA of interest and whether that miRNA is dysregulated in response to genetic susceptibility gene possession or triggered by ongoing immune responses during the course of SLE progression. To determine when miRNA therapeutics would be most effective, miRNA dampening or loss of miRNAs at specific stages of disease progression in mouse models can begin to clarify therapeutic approaches and effectiveness through different stages of disease development. Lastly, while those miRNAs that produce strong effects in mouse models are of obvious consideration for targeting, miRNA redundancy does exist. Targeting multiple miRNAs, or miRNA families with conserved target sequences, may produce additive effects compared to single miRNA targeting. After weighing both the advantages and possible unintended consequences in the development of miRNA therapeutics, targeting miRNAs can be deemed as a promising option, but much proof of concept and testing remains to be addressed.

## Conclusions

The studies reviewed herein demonstrate the essential roles of miRNAs in B cell responses and the development of autoimmune disease. Not only do miRNAs promote these responses, but some miRNAs also possess critical negative regulatory functions. While the studies to date have begun to elucidate the role of these factors, much work remains to fully characterize their impact during autoimmunity, as well as the capacity to effectively target them to dampen disease. Additionally, many miRNAs have yet to be studied using *in vivo* mouse systems but with increasing access to mouse models, the list of miRNAs involved in the processes discussed herein will likely expand significantly. Extensive miRNA expression profiling in cell types and specific stages of the B cell response will aid in identifying additional miRNAs worth studying in mouse models in the future. While simply identifying the miRNAs and their target genes that contribute to disease is important, further attention to the level of miRNA expression that modulates the observed effects and the stage of disease at which the miRNA of interest is involved has important therapeutic ramifications that must be addressed more heavily in future studies. Lastly, traditional miRNA mediated regulation has been of focus, but non-traditional regulation by miRNAs is an interesting avenue of research that deserves significant attention and may alter how we approach targeting miRNA function. Altogether, the further study and modeling of miRNAs in relevant animal systems will inform future therapeutic design and identify those miRNAs that may provide the most promising targets for potential miRNA-centric therapeutic development to treat SLE.

## Author Contributions

SS and ZR wrote the article. SS designed figures. All authors contributed to the article and approved the submitted version.

## Funding

This work was supported by National Institutes of Health R21AI128111 to ZR.

## Conflict of Interest 

The authors declare that the research was conducted in the absence of any commercial or financial relationships that could be construed as a potential conflict of interest.
